# Systematic Exploration of Functional Group Relevance for Anti-Leishmanial Activity of Anisomycin

**DOI:** 10.3390/biomedicines11092541

**Published:** 2023-09-15

**Authors:** Anh Minh Thao Nguyen, Moran Shalev-Benami, Chloé Rosa-Teijeiro, Ana Victoria Ibarra-Meneses, Ada Yonath, Anat Bashan, Charles L. Jaffe, Martin Olivier, Christopher Fernandez-Prada, William D. Lubell

**Affiliations:** 1 Department of Chemistry, Université de Montréal, Montreal, QC H3T 1J4, Canada; 2 Department of Chemical and Structural Biology, Weizmann Institute of Science, Rehovot 7610001, Israel; moransb@weizmann.ac.il (M.S.-B.); ada.yonath@weizmann.ac.il (A.Y.); anat.bashan@weizmann.ac.il (A.B.); 3 Department of Pathology and Microbiology, Faculty of Veterinary Medicine, Université de Montréal, Saint-Hyacinthe, QC J2S 2M2, Canada; chloe.rosa-teijeiro@umontreal.ca (C.R.-T.); ana.victoria.ibarra.meneses@umontreal.ca (A.V.I.-M.); christopher.fernandez.prada@umontreal.ca (C.F.-P.); 4 The Research Group on Infectious Diseases in Production Animals (GREMIP), Faculty of Veterinary Medicine, Université de Montréal, Montreal, QC J2S 2M2, Canada; 5 Department of Microbiology & Molecular Genetics, Kuvin Center for the Study of Tropical & Infectious Diseases, Institute for Medical Research (IMRIC), Hadassah Hebrew University Medical Center, Jerusalem 9112102, Israel; cjaffe@mail.huji.ac.il; 6 Departments of Medicine, and of Microbiology and Immunology, Faculty of Medicine, McGill University, Montreal, QC H3A 2B4, Canada; martin.olivier@mcgill.ca; 7 The Research Institute of the McGill University Health Centre (RI-MUHC), Montreal, QC H4A 3J1, Canada

**Keywords:** anisomycin, *Leishmania*, ribosome

## Abstract

Assessment of structure–activity relationships for anti-protozoan activity revealed a strategy for preparing potent anisomycin derivatives with reduced host toxicity. Thirteen anisomycin analogs were synthesized by modifying the alcohol, amine, and aromatic functional groups. Examination of anti-protozoal activity against various strains of *Leishmania* and cytotoxicity against leucocytes with comparison against the parent natural product demonstrated typical losses of activity with modifications of the alcohol, amine, and aromatic meta-positions. On the other hand, the para-phenol moiety of anisomycin proved an effective location for introducing substituents without significant loss of anti-protozoan potency. An entry point for differentiating activity against *Leishmania* versus host has been uncovered by this systematic study.

## 1. Introduction

Anisomycin (**1**) is an antibiotic isolated from Streptomyces [1]. First demonstrated to inhibit protein translation with eukaryote selectivity [2], anisomycin has since been shown to target various organisms by multiple mechanisms of action [3]. In complex with the 80S ribosome, anisomycin reorients the 25S rRNA residue 2397 (2055) influencing the conformation of the nearby U2873 (U2504) residue and preventing aminoacyl-tRNA binding to the peptidyl transferase site [4]. The 2397 (2055) rRNA nucleotide has been suggested to dictate binding selectivity between bacterial- and eukaryotic-specific inhibitors because cytosine and adenine are typically found, respectively, in bacteria and most eukaryotes [4]. At concentrations below those inhibitive of protein synthesis, anisomycin elicited specific and strong activation of mitogen-activated protein kinases (MAPKs, e.g., JNK and p38) and induced c-fos gene expression [5,6]. Initiation of p38 MAP kinase followed by inhibition of protein synthesis by anisomycin in mammalian cells was shown to lead to activation of the glucose transporter GLUT1, an early event in the adaptive response of mammalian cells to metabolic stress [7].

Anisomycin has exhibited activity against protozoa and viruses, as well as mammalian macrophages and cancer cells. Activation of p38 signaling by anisomycin reduced *L. donovani* survival within human macrophages in vitro by a mechanism blocked by SB203580, a p38-specific inhibitor [8]. Anisomycin reversed Japanese encephalitis virus-induced down-regulation of extracellular signal-regulated protein kinase (ERK) phosphorylation and protected murine neuroblastoma (N18) cells against infection [9]. The anti-viral activity of anisomycin was further demonstrated against dengue virus (DENV) and Zika virus (ZIKV) strains in Vero cells by a mode of action implicating likely blockage of macromolecular synthesis instead of p38 MAPK activation. In ZIKV-infected mice, a low dose of anisomycin caused a significant reduction in viral levels and enhanced survival [10]. In rabbit atheroma-like lesions in vivo, anisomycin decreased selective macrophage content by a mechanism implicating p38 MAPK [11]. In addition, as an inducer of apoptosis in tumor cells, anisomycin inhibited angiogenesis, proliferation, and invasion in ovarian cancer cells [12]. Moreover, anisomycin inhibited T cell behavior, curbing transplantation rejection without significant side effects at effective therapeutic doses; however, over-dosage led to pulmo-, nephro-, and hepato-toxicity, slight micronucleus formation, and sperm aberration [13].

**Figure 1:**
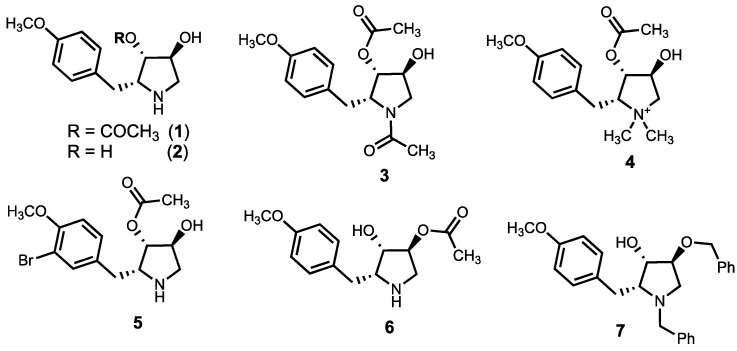
Anisomycin (**1**) and derivatives **2**–**7**.

Interest in anisomycin has led to various syntheses of the densely functionalized chiral pyrrolidine structure and derivatives using a variety of starting materials, such as various carbohydrate and amino acid precursors [14]. Early studies of relationships between anisomycin structure and activity on protein translation demonstrated that removal of the 3-position acetoxy group (desacetylanisomycin, **2**), nitrogen acetylation and quaternization, as well as aromatic bromination, all resulted in analogs (e.g., **3**–**5**) exhibiting significant reductions of activity [2]. Movement of the acetyl group from the 3- to the 4-position alcohol gave isomer **6**, which exhibited 3-fold less inhibitory activity on protein synthesis but comparable cytotoxicity as anisomycin in four tumor cell lines [15]. Activity against protozoa and fungi dropped mildly on replacement of the para-methoxy substituent by a methyl group or proton but was significantly reduced on moving the methoxy group from the para to the meta and ortho positions, as well as upon replacement of the benzyl methylene protons by methyl and phenyl substituents [16]. In the triple-negative breast cancer cell line MDA-MB-468, both anisomycin and (2*R*,3*S*,4*S*)-1-benzyl-4-benzyloxy-3-hydroxy-2-(4-methoxybenzyl)-pyrrolidine (**7**) inhibited protein synthesis and exhibited cytotoxicity at equal concentrations [17]. In examinations of their ability to activate, respectively, JNK and p38 MAPK, replacement of the (4*S*)-alcohol by hydrogen or a methyl group gave analogs exhibiting similar and significantly reduced activity relative to anisomycin and desacetylanisomycin (**2**); however, *N*- and *O*-benzyl analogs (e.g., **7**) did not exhibit kinase activating activity [18]. The combination of *N*-acylation in various amide, carbamate, and urea components and replacement of the acetyl group by different carbamoyl residues gave a library of anisomycin analogs exhibiting activity against *Staphylococcus aureus* and *Candida albicans* in whole-cell assays, with reduced cytotoxicity against HEK293 mammalian cells [19]. In sum, the structure–activity data for anisomycin is relatively dispersed and somewhat challenging to interpret due in part to the range of targeted indications and mechanisms of action, the latter of which include inhibitory activity on protein synthesis and the potential to activate various kinases (Figure 1). 

Possessing broad-spectrum anti-parasitic activity [20], anisomycin has shown activity against leishmaniasis [21]. A parasitic disease infecting 12 to 15 million people worldwide, leishmaniasis causes more than 1 million new cases annually [22]. Presenting in four main forms (e.g., cutaneous (CL), mucocutaneous (MCL), visceral (VL), and post Kala-azar dermal (PKDL)) [22], leishmaniasis infects the spleen, liver, and bone marrow [23], causes skin lesions [24], and destroys mucous membranes [25]. New safe anti-leishmanial agents are urgently needed because host toxicity and resistance have limited the efficacy of the current arsenal of drugs used to treat *Leishmania*, which includes pentavalent antimonial, amphotericin B, miltefosine, and paromomycin [26,27]. 

In the interest of furthering the utility of anisomycin for treating *Leishmania*, a systematic structure–activity relationship study has been performed to identify positions for modification to ideally improve selectivity by increasing anti-parasite activity and reducing toxicity. Modifications were performed on the secondary amine, hydroxyl, and acetyl groups. Moreover, the aromatic ring was modified at the meta- and para-positions. Although the structural elements for maintaining activity were illustrated, modifications were typically unfruitful in providing active and selective analogs. Replacement of the methoxy group by other ethers was, however, found to offer a point of entry for altering anisomycin in a way that could maintain activity yet improve anti-protozoan selectivity. 

## 2. Materials and Methods

### 2.1. In Vitro Inhibition of Translation Assays

Cytosolic ribosome susceptibility and selectivity were assessed through three different cell-free transcription-translation assays: (1) An extract of *E. coli* (S30, Promega, Madison, WI, USA), (2) in rabbit reticulocytes (Promega), and (3) in *L. tarentolae* lysate (Jena Bioscience, Jena, Germany). Three plasmids, compatible with each extract system, were used in this study: pBESTlucTM (Promega) for the prokaryotic translation assay, Luciferase T7 DNA (Promega) for the reticulocytes, and pLEXSY-invitro2-EGFP (Jena Bioscience) for *Leishmania*. Firefly luciferase was used as a reporter gene for the bacterial and reticulocyte systems, and EGFP for *Leishmania*. Reaction mixtures were prepared as suggested by the manufacturer, except that the final reaction volume was adjusted to 15 μL to which 1.5 μL (ten-fold) of the relevant compound concentration was supplemented. Assays were performed in white polystyrene 96-well flat-bottom plates (Nunc, Roskilde, Denmark) for luciferase and black polystyrene 384-well flat-bottom plates (Greiner, Kremsmunster, Austria) for EGFP. Incubation times were 60 min at 37 °C, 90 min at 30 °C, and 120 min at 26 °C for the bacterial, reticulocyte, and *Leishmania* systems, respectively. Reactions were stopped by quick snap cooling followed by a 5 min incubation on ice. Luciferase activity was measured for each well following the addition of 50 μL of Luciferase Assay reagent (Promega, Madison, WI, USA) with an automatic reagent injector (Tecan, Mannedorf, Switzerland) by recording the chemical-luminescence signal. EGFP fluorescence (λex = 488 nm; λem = 507 nm) was recorded on a Tecan Infinite R^®^F200 microplate reader (Tecan). Extracts lacking the circular DNA template were used as a negative control to calculate the fluorescence/chemical luminescence background. Reaction mixtures without compounds were used as positive controls and were regarded as 100% translation. Paromomycin was used as a control reagent in all assays. At least six different concentrations were used to plot each translation inhibition curve; experiments were performed in three independent repeats in duplicate. Half maximal inhibition concentrations (IC_50_) values were calculated from the concentration-response fitting curves using GraFit7.0.3 software [28].

### 2.2. Leishmania Cultures and Anti-Leishmanial Activity Determination

The *L. infantum* (MHOM/MA/67/ITMAP-263) wild-type strain (WT), as well as the resistant mutants Sb2000.1, AmB1000.1, and MF200.5 [29,30,31,32,33], which are respectively resistant to antimony (Sb^III^), amphotericin B (AmB), and miltefosine (MF), were grown in M199 medium at 25 °C supplemented with 10% fetal bovine serum, 5 μg/mL of haemin at pH 7.0, 2000 μM Sb (Potassium antimonyl tartrate trihydrate, Sigma-Aldrich, Saint Louis, MO, USA), 200 μM of MF (Miltefosine, Cayman Chem, Ann Arbor, MI, USA) or 1 μM AmB (Amphotericin B solution, Sigma-Aldrich, Saint Louis, MO, USA). Leishmanicidal values were determined in *Leishmania* promastigotes by monitoring the replication of parasites after 72 h of incubation at 25 °C in the presence of increasing concentrations of the different compounds by measuring A_600_ using a Cytation 5 machine (BioTek-Agilent, Santa Clara, CA 9, USA). EC_50_ values were calculated based on dose–response curves analyzed by non-linear regression with GraphPad Prism 10.0 software (GraphPad Software, La Jolla, CA, USA). An average of at least three independent biological replicates were performed for each determination. 

### 2.3. Drug Susceptibility Assays in Infected BMDM

The effect of isopropyl ether **22a** on the amastigote intracellular form of *Leishmania* was determined by the measurement of EC_50_ values in cells, as previously described [34]. Briefly, 2.5 × 10^5^ bone-marrow-derived macrophages (BMDMs) were plated into the wells of 12-well chamber slides (Ibidi) with complete DMEM medium. A culture of *L. infantum* or *L. major* WT promastigotes in stationary phase in a ratio of 1:5 BMDM to parasites was used to infect cells. Infection was carried out for 3 h at 37 °C in DMEM medium with 5% CO_2_ without drug. After 24 h, increasing concentrations of ether **22a** were added to the medium containing the infected macrophages. After 48 h, the slides were fixed in methanol and stained with Diff-Quick solution to facilitate parasite visualization. The number of infecting amastigotes per 100 cells was determined by examining 300 macrophages per triplicate assay and normalized to the untreated control. EC_50_ values in the amastigote form were calculated based on dose–response curves that were analyzed by non-linear regression using the GraphPad Prism 9.0 software. An average of three independent biological replicates was used to perform the analyses.

### 2.4. Superimposition of Ribosome Structures

*L. major* 80S ribosome (8OVJ) was superimposed on the *H. marismortui* 50S ribosome (PDBID: 3CC4) in COOT [35], and figures were prepared using UCSF ChimeraX [36]. The identity of RNA modifications in *L. major* was derived from the genome-wide analysis [37].

### 2.5. Chemistry

Anhydrous solvents (THF, DMF, MeCN) were obtained by passage through solvent filtration systems (GlassContour, Irvine, CA, USA). Deionized water was used in the purifications. Unless specified otherwise, all reagents were from commercial sources and used as received. Anisomycin and di-*tert*-butyl decarbonate (Boc_2_O) were purchased from ChemImpex; acetic anhydride was purchased from Alfa Aesar; pyridine, 4-dimethylaminopyridine (DMAP), sodium hydride, iodomethane, boron tribromide, trifluoroacetic acid (TFA), *N*-iodosuccinimide (NIS), dimethylamine (40% wt. in water), formaldehyde (37% wt. in water), *N*-methylmorpholine, cesium carbonate, 2-iodopropane, 1-iodooctane, benzyl bromide, *N*,*N*-dimethylethylenediamine, formic acid (FA), and triethylamine, all were purchased from Sigma-Aldrich; acetic acid, sodium hydroxide, potassium carbonate and solvents were obtained from Fisher. Sodium hydride (60% dispersion in mineral oil) was washed with hexane three times to remove oil prior to use. Chromatography was on 230−400 mesh silica gel. Analytical thin-layer chromatography (TLC) was performed on glass-backed silica gel plates (Merck 60 F254). Visualization of the developed chromatogram was performed by UV absorbance or staining with ninhydrin. ^1^H and ^13^C NMR spectra were measured respectively in CD_3_OD at 500 MHz and 126 MHz and referenced to CD_3_OD (3.31 ppm and 49.0 ppm). Coupling constant *J* values were measured in Hertz (Hz) and chemical shift values in parts per million (ppm). Specific rotations, [α]_D_ were measured at 25 °C at the specified concentrations (*c* in g/100 mL) using a 1 dm cell on a PerkinElmer Polarimeter 589 and expressed using the general formula: ${\left[\alpha \right]}_{25}^{D}$ = (100 × α)/(d × c). High resolution mass spectrometric analyses were obtained by the Centre Régional de Spectrométrie de Masse de l’Université de Montréal. Protonated molecular ions [M + H]^+^ and sodium adducts [M + Na]^+^ were used for empirical formula confirmation. 

#### 2.5.1. (2*R*,3*S*,4*S*)-3,4-dihydroxy-2-(4-methoxybenzyl)-pyrrolidine (**2**)

A mixture of anisomycin (**1**, 10 mg, 0.038 mmol, 1 eq.) and NaOH 1N (0.25 mL) was heated and stirred for 3 h at 80 °C. After cooling to room temperature, the resulting mixture was extracted with ethyl acetate. The organic layer was washed with brine, dried over MgSO_4_, filtered, and evaporated. The residue was purified by HPLC on a C18 column using 5 to 50% MeOH in H_2_O. Evaporation of the collected fractions provided diol **2** as white solid (5.9 mg, 70%), which was shown to be >99% pure by LC-MS analysis [5−50% MeOH (0.1% FA) in H_2_O (0.1% FA) over 14 min, RT 3.87 min]: [α]_D_^25^ −23.7 (*c* 0.49, MeOH); ^1^H NMR (400 MHz, MeOD) δ 7.26 (d, *J* = 8.7 Hz, 2H), 6.91 (d, *J* = 8.7 Hz, 2H), 4.25 (d, *J* = 4.0 Hz, 1H), 3.97 (s, 1H), 3.86 (td, *J* = 8.2, 2.8 Hz, 1H), 3.77 (s, 3H), 3.60 (dd, *J* = 12.4, 4.4 Hz, 1H), 3.18–3.05 (m, 2H), 2.94 (dd, *J* = 14.1, 8.3 Hz, 1H). ^13^C NMR (101 MHz, MeOD) δ 160.4, 131.1, 130.0, 115.3, 76.1, 75.8, 65.3, 55.7, 52.6, 32.2. HRMS (ESI^+^) calcd *m*/*z* for C_12_H_18_NO_3_ [M + H]^+^ 224.1281, found 224.1280.

#### 2.5.2. (2*R*,3*S*,4*S*)-1-methyl-4-hydroxy-2-(4-methoxybenzyl)-pyrrolidin-3-yl acetate (**8**)

A mixture of anisomycin (**1**, 0.038 mmol, 10 mg, 1 eq.), formaldehyde (0.2 mL, 37% in water), and formic acid (0.2 mL) in a sealed tube was heated with microwave irradiation at 80 °C for 6 h. After cooling to room temperature, the reaction mixture was extracted with EtOAc (3 × 5 mL). The organic phase was combined, washed with aq. saturated NaHCO_3_ (5 mL) and brine (5 mL), dried over MgSO_4_, filtered, and concentrated in vacuo. The residue was purified by HPLC on a C18 column using a gradient of 30% to 90% MeOH (0.1% FA) in H_2_O (0.1% FA) to give *N*-methyl pyrrolidine **8** (6.2 mg, 58%), which was shown to be >99% pure by LC-MS analysis [40−90% MeOH (0.1% FA) in H_2_O (0.1% FA) over 14 min, RT 4.56 min]: [α]_D_^25^ 54.7 (*c* 0.52, MeOH); ^1^H NMR (400 MHz, MeOD) δ 8.45 (s, 1H), 7.16 (d, *J* = 8.7 Hz, 2H), 6.88 (d, *J* = 8.7 Hz, 2H), 4.17 (d, *J* = 5.2 Hz, 1H), 3.82–3.78 (m, 1H), 3.77 (s, 3H), 3.61–3.53 (m, *J* = 5.2 Hz, 1H), 3.07 (dd, *J* = 13.5, 5.9 Hz, 1H), 2.87 (dd, *J* = 13.5, 9.9 Hz, 1H), 2.78 (d, *J* = 12.1 Hz, 1H), 2.73 (s, 3H), 2.15 (s, 3H). ^13^C NMR (101 MHz, MeOD) δ 171.2, 160.3, 130.9, 115.3, 80.0, 73.7, 71.4, 63.9, 55.7, 42.6, 31.8, 30.4, 20.7. HRMS (ESI^+^) calcd *m*/*z* for C_15_H_21_NO_4_ [M^+^], 279.1471, found 279.1487.

#### 2.5.3. (2*R*,3*S*,4*S*)-3,4-diacetoxy-2-(4-methoxybenzyl)-pyrrolidine hydrochloride (**9**)

A solution of anisomycin (**1**, 10 mg, 0.038 mmol, 1 eq.) in THF (0.2 mL) at room temperature was treated with Boc_2_O (9.1 mg, 0.042 mmol, 1.1 eq.), stirred for 2 h, and concentrated under reduced pressure to give a colorless liquid (14.1 mg), which was dissolved in pyridine (0.2 mL), cooled to 0 °C, and treated with Ac_2_O (3.95 μL, 0.042 mmol, 1.1 eq.) and DMAP (0.5 mg, 0.1 eq.). After stirring overnight, the mixture warmed to room temperature. The volatiles were removed under reduced pressure. The residue was dissolved in EtOAc (5 mL), washed with 10% aq. HCl (2 × 2 mL), brine (2 mL), and dried over MgSO_4_. The organic phase was concentrated in vacuo and purified by flash chromatography on silica gel eluting with a gradient of 50–90% EtOAc in hexane. Evaporation of the collected fractions gave a colorless liquid [9.9 mg, Rf = 0.56 (60% EtOAc in hexane)], which was dissolved in DCM (2 mL) and treated with HCl gas bubbles for 1 h. The reaction mixture was evaporated under reduced pressure to give hydrochloride **9** (8.5 mg, 66%). The product was shown to be >95% pure by LC-MS analysis [30−90% MeOH (0.1% FA) in H_2_O (0.1% FA) over 14 min, RT 7.86 min]: [α]_D_^25^ 26.2 (*c* 0.72, MeOH); ^1^H NMR (400 MHz, MeOD) δ 7.25 (d, *J* = 8.4 Hz, 2H), 6.93 (d, *J* = 8.3 Hz, 2H), 5.29 (dd, *J* = 14.9, 3.3 Hz, 2H), 4.12 (br s, 1H), 3.85–3.79 (m, 1H), 3.78 (s, 3H), 3.37 (d, *J* = 13.7 Hz, 1H), 3.11 (dd, *J* = 14.3, 5.7 Hz, 1H), 2.96 (dd, *J* = 14.2, 9.0 Hz, 1H), 2.21 (s, 3H), 2.11 (s, 3H). ^13^C NMR (101 MHz, MeOD) δ 170.5, 170.2, 160.5, 130.8, 128.3, 115.4, 75.6, 75.3, 64.3, 55.6, 50.3, 31.9, 20.5, 20.4. HRMS (ESI^+^) calcd *m*/*z* for C_16_H_22_NO_5_ [M + H]^+^ 308.1492, found 308.1500.

#### 2.5.4. (2*R*,3*S*,4*S*)-3,4-dimethoxy-2-(4-methoxybenzyl)-pyrrolidine hydrochloride (**10**)

A solution of anisomycin (**1**, 10 mg, 0.038 mmol, 1 eq.) in THF (0.2 mL) at room temperature was treated with Boc_2_O (9.1 mg, 0.042 mmol, 1.1 eq.), stirred for 2 h, and concentrated under reduced pressure to give Boc-anisomycin **12** (14.1 mg), which was dissolved in MeOH (3 mL) at 0 °C and treated with K_2_CO_3_ (5.8 mg, 0.042 mmol, 1.1 eq.) and stirred for 30 min. The volatiles were removed under reduced pressure. The residue was dissolved in EtOAc (5 mL), washed with brine (2 × 3 mL), dried over MgSO_4,_ filtered, and concentrated in vacuo to give a residue [11.9 mg, Rf = 0.38 (60% EtOAc in hexane)]. The residue (11.9 mg, 0.037 mmol, 1 eq.) was dissolved in DMF (2 mL), cooled to 0 °C, and treated with NaH (2.2 mg, 0.084 mmol, 2.2 eq.), followed by iodomethane (5.2 μL, 0.084 mmol, 2.2 eq.). After stirring for 3 h, the reaction mixture, which had warmed to room temperature, was cooled to 0 °C and treated with water. The reaction mixture was extracted with EtOAc (3 × 5 mL). The organic layers were combined, washed with brine (5 mL), dried over MgSO_4_, filtered, and evaporated to a residue, which was purified by flash chromatography on silica gel eluting with a gradient of 50–90% EtOAc in hexane. Evaporation of the collected fractions gave a colorless liquid [11.5 mg, Rf = 0.46 (30% EtOAc in hexane)], which was dissolved in DCM (2 mL) and treated with HCl gas bubbles for 1 h. Evaporation of the reaction mixture gave 3,4-dimethoxy-pyrrolidine **10** (6.9 mg, 67%), which was shown to be >95% pure by LC-MS analysis [40−90% MeOH (0.1% FA) in H_2_O (0.1% FA) over 14 min, RT 7.22 min]: [α]_D_^25^ − 38.4 (*c* 0.57, MeOH); ^1^H NMR (400 MHz, MeOD) δ 7.24 (d, *J* = 8.7 Hz, 2H), 6.92 (d, *J* = 8.7 Hz, 2H), 4.14 (d, *J* = 4.6 Hz, 1H), 3.85–3.79 (m, *J* = 8.0, 3.9 Hz, 1H), 3.78 (s, 3H), 3.74 (d, *J* = 3.1 Hz, 1H), 3.54 (dd, *J* = 13.0, 4.5 Hz, 1H), 3.48 (s, 3H), 3.37 (s, 3H), 3.27 (d, *J* = 13.0 Hz, 1H), 3.13 (dd, *J* = 13.9, 7.4 Hz, 1H), 2.96 (dd, *J* = 13.9, 8.1 Hz, 1H). ^13^C NMR (101 MHz, MeOD) δ 160.4, 131.1, 129.5, 115.4, 82.4, 81.0, 65.0, 58.1, 57.5, 55.7, 50.0, 31.9. HRMS (ESI^+^) calcd *m*/*z* for C_14_H_22_NO_3_ [M + H]^+^ 252.2594, found 252.1599.

#### 2.5.5. (2*R*,3*S*,4*S*)-3-methoxy-4-hydroxy-2-(4-methoxybenzyl)-pyrrolidine hydrochloride (**11**)

Anisomycin (**1**, 10 mg, 0.038 mmol, 1 eq.) in MeCN (0.2 mL) was treated with Boc_2_O (16.5 mg, 0.076 mmol, 2 eq.) and DMAP (0.5 mg, 0.004 mmol, 0.1 eq.) and stirred at room temperature for 20 h. Evaporation of the mixture under reduced pressure gave bis-Boc-anisomycin **14** (18.1 mg), which was dissolved in MeOH (3 mL), cooled to 0 °C, treated with K_2_CO_3_ (5.8 mg, 0.042 mmol, 1.1 eq.), stirred for 30 min, and evaporated under reduced pressure. The residue was dissolved in EtOAc (5 mL), washed with brine (2 × 3 mL), dried over MgSO_4_, filtered, and concentrated in vacuo to a residue. The residue was dissolved in DMF (2 mL), cooled to 0 °C, and treated with NaH (1.09 mg, 0.046 mmol, 1.2 eq.) and MeI (2.83 μL, 0.046 mmol, 1.2 eq.). After stirring for 3 h, the mixture warmed to room temperature, was cooled to 0 °C, and was treated with water. The mixture was extracted with EtOAc (3 × 5 mL). The organic layers were combined, washed with brine (5 mL), dried over MgSO_4_, filtered, and evaporated to a residue, which was purified by flash chromatography on silica gel eluting with a gradient of 50–90% EtOAc in hexane. Evaporation of the collected fractions gave a colorless liquid [9.8 mg, Rf = 0.34 (30% EtOAc in hexane)], which was dissolved in DCM (2 mL) and treated with HCl gas bubbles for 5 h. Evaporation of the volatiles under reduced pressure gave methyl ether **11** (6.1 mg, 59%), which was shown to be >95% pure by LC-MS analysis [40−90% MeOH (0.1% FA) in H_2_O (0.1% FA) over 14 min, RT 5.78 min]: [α]_D_^25^ −45 (*c* 0.44, MeOH); ^1^H NMR (400 MHz, MeOD) δ 7.23 (d, *J* = 8.6 Hz, 2H), 6.92 (d, *J* = 8.7 Hz, 2H), 4.49 (d, *J* = 4.4 Hz, 1H), 3.99–3.90 (m, 1H), 3.78 (s, 3H), 3.58–3.52 (m, 2H), 3.46 (s, 3H), 3.12 (dd, *J* = 13.1, 6.9 Hz, 2H), 2.96 (dd, *J* = 13.8, 7.9 Hz, 1H). ^13^C NMR (101 MHz, MeOD) δ 160.4, 131.0, 129.7, 115.4, 85.2, 71.4, 64.7, 58.1, 55.7, 52.7, 31.0. HRMS (ESI^+^) calcd *m*/*z* for C_13_H_20_NO_3_ [M + H]^+^ 238.1438, found 238.1446.

#### 2.5.6. (2*R*,3*S*,4*S*)-4-hydroxy-2-(3-iodo-4-methoxybenzyl)-pyrrolidin-3-yl acetate (**16**)

A solution of anisomycin (**1**, 10 mg, 0.038 mmol, 1 eq.) in DCM (2 mL) was treated with trifluoroacetic acid (2.9 μL, 0.046 mmol, 1.2 eq.) and *N*-iodosuccinimide (10.2 mg, 0.046 mmol, 1.2 eq.), stirred for 4 h, and concentrated to a residue, which was purified by HPLC on a C18 column using a gradient of 5% to 50% MeCN (0.1% FA) in H_2_O (0.1% FA). Evaporation of the collected fractions gave *o*-iodobenzyl pyrrolidine **16** (7.8 mg, 53%), which was shown to be >99% pure by LC-MS analysis [5−50% MeCN (0.1% FA) in H_2_O (0.1% FA) over 14 min, RT 4.93 min]: [α]_D_^25^ 27.6 (*c* 0.34, MeOH); ^1^H NMR (500 MHz, MeOD) δ 8.52 (s, 1H), 7.74 (d, *J* = 1.9 Hz, 1H), 7.29 (dd, *J* = 8.4, 1.9 Hz, 1H), 6.93 (d, *J* = 84 Hz, 1H), 5.02 (s, 1H), 4.32 (d, *J* = 4.3 Hz, 1H), 4.13–4.06 (m, 1H), 3.85 (s, 3H), 3.56 (dd, *J* = 12.6, 3.9 Hz, 1H), 3.15 (d, *J* = 12.6 Hz, 1H), 3.02 (dd, *J* = 14.2, 7.0 Hz, 1H), 2.98–2.90 (m, 1H), 2.18 (s, 3H); ^13^C NMR (126 MHz, MeOD) δ 170.9, 159.1, 140.9, 131.6, 131.2, 112.4, 86.8, 78.6, 73.8, 63.1, 56.9, 52.6, 31.9, 20.7. HRMS (ESI^+^) calcd *m*/*z* for C_14_H_19_INO_4_ [M + H]^+^ 392.0353, found 392.0346.

#### 2.5.7. (2*R*,3*S*,4*S*)-4-hydroxy-2-(4-hydroxybenzyl)-pyrrolidin-3-yl acetate (**17**)

A solution of anisomycin (**1**, 10 mg, 0.038 mmol, 1 eq.) in DCM (3 mL) at –78 °C was treated with BBr_3_ (0.06 mL, 0.057 mmol, 1.5 eq., 1 M in DCM). The ice bath was removed. After stirring for 2.5 h, the reaction mixture warmed to room temperature and was concentrated under reduced pressure. The residue was purified by HPLC on a polar-RP column using a gradient of 20 to 80% MeOH (0.1% FA) in H_2_O (0.1% FA) to give hydroxybenzyl pyrrolidine **17** (5.34 mg, 56%), which was shown to be >99% pure by LC-MS analysis [20−80% MeOH (0.1% FA) in H_2_O (0.1% FA) over 14 min, RT 5.69 min]: [α]_D_^25^ 20.3 (*c* 0.18, MeOH); ^1^H NMR (700 MHz, MeOD) δ 8.52 (br s, 1H), 7.11 (d, *J* = 8.4 Hz, 2H), 6.77 (d, *J* = 8.5 Hz, 2H), 5.03 (br s, 1H), 4.33 (d, *J* = 4.3 Hz, 1H), 4.09 (d, *J* = 6.9 Hz, 1H), 3.57 (dd, *J* = 12.7, 4.5 Hz, 1H), 3.16 (d, *J* = 12.7 Hz, 1H), 3.02 (dd, *J* = 14.2, 6.9 Hz, 1H), 2.92 (dd, *J* = 14.2, 8.6 Hz, 1H), 2.17 (s, 3H); ^13^C NMR (176 MHz, MeOD) δ 170.9, 157.9, 130.9, 127.9, 116.8, 78.5, 73.6, 63.6, 52.6, 32.4, 20.6. HRMS (ESI^+^) calcd *m*/*z* for C_13_H_18_NO_4_ [M + H]^+^ 252.1230, found 252.1223.

#### 2.5.8. (2*R*,3*S*,4*S*)-4-hydroxy-2-[3,5-di-(dimethylaminomethyl)-4-hydroxybenzyl]-pyrrolidin-3-yl acetate (**19**)

A mixture of hydroxybenzyl pyrrolidine **17** (91% pure, 9.61 mg, 0.038 mmol, 1 eq.) and aq. saturated NaHCO_3_ (0.2 mL) in THF (3 mL) at pH = 8–9 was treated with Boc_2_O (9.1 mg, 0.042 mmol, 1.1 eq.), stirred for 1 h, and concentrated in vacuo. The reduced volume was partitioned between EtOAc (5 mL) and water (2 mL). The organic phase was dried over MgSO_4_, filtered, and evaporated in vacuo to a residue, which was purified by flash chromatography on silica gel eluting with a gradient of 60–90% EtOAc in hexane. Evaporation of the volatiles gave Boc-desmethylanisomycin **18** (7.90 mg, 59%), which was dissolved in glacial acetic acid (2 mL) and treated with dimethylamine (6.1 μL, 0.044 mmol, 2.2 eq., 40 wt.% in H_2_O) and formaldehyde (7.9 μL, 0.044 mmol, 2.2 eq., 37% in water), heated at 50 °C, stirred for 24 h, and concentrated in vacuo. The reduced volume was extracted with EtOAc (2 × 5 mL). The organic layers were combined, washed with brine (5 mL), dried over MgSO_4_, filtered, and evaporated to a residue. The residue was dissolved in DCM (5 mL) and treated with HCl gas bubbles for 1 h. The volatiles were removed under reduced pressure. The residue was purified by HPLC on a polar-RP column using a gradient of 20% to 80% MeOH (0.1% FA) in H_2_O (0.1% FA) to give pyrrolidine **19** (5.3 mg, 38%), which was shown to be >99% pure by LC-MS analysis [20−80% MeOH (0.1% FA) in H_2_O (0.1% FA) over 14 min, RT 3.28 min]: [α]_D_^25^ 18.5 (*c* 0.28, MeOH); ^1^H NMR (500 MHz, MeOD) δ 7.61 (s, 2H), 5.49 (s, 1H), 5.19 (d, *J* = 3.0 Hz, 1H), 4.45 (s, 4H), 4.38 (d, *J* = 4.6 Hz, 1H), 4.31–4.27 (m, 1H), 3.66 (dd, *J* = 12.7, 4.4 Hz, 1H), 3.26 (d, *J* = 12.7 Hz, 1H), 3.18–3.08 (m, 1H), 2.92 (s, 12H), 2.21 (s, 3H). ^13^C NMR (126 MHz, MeOD) δ 171.0, 156.0, 137.0, 130.7, 121.2, 78.3, 73.5, 63.2, 57.3, 54.8, 52.7, 43.4, 31.8, 20.8. HRMS (ESI^+^) calcd *m*/*z* for C_19_H_32_N_3_O_4_ [M + H]^+^ 366.2387, found 366.2379.

#### 2.5.9. (2*R*,3*S*,4*S*)-4-hydroxy-2-[3,5-di-(morpholinomethyl)-4-hydroxybenzyl]-pyrrolidin-3-yl acetate (**20**)

Employing the protocol for the synthesis of Mannich adduct pyrrolidine **19**, the residue (8.2 mg, 0.023 mmol, 1 eq.) from treatment of hydroxybenzyl pyrrolidine **17** with Boc_2_O, was dissolved in glacial acetic acid (2 mL) and treated with *N*-morpholine (4.1 μL, 0.046 mmol, 2.2 eq.) and formaldehyde (8.3 μL, 0.046 mmol, 2.2 eq., 37% in water). After heating at 50 °C and stirring for 20 h, the mixture was worked up and treated as described above. The residue was purified by HPLC on a polar-RP column using a gradient of 20% to 80% MeOH in H_2_O to give morpholine **20** (4.2 mg, 41%), which was shown to be >99% pure by LC-MS analysis [20−80% MeOH (0.1% FA) in H_2_O (0.1% FA) over 14 min, RT 3.52 min]: [α]_D_^25^ 15.2 (*c* 0.29, MeOH); ^1^H NMR (500 MHz, MeOD) δ 7.69 (s, 2H), 5.22 (d, *J* = 2.6 Hz, 1H), 4.48 (s, 4H), 4.39 (d, *J* = 4.4 Hz, 1H), 4.34–4.27 (m, 1H), 4.09–4.00 (m, 4H), 3.91 (m, 4H), 3.66 (dd, *J* = 12.7, 4.4 Hz, 1H), 3.56–3.49 (m, 4H), 3.33–3.24 (m, 5H), 3.17 (dd, *J* = 14.7, 5.5 Hz, 1H), 3.09 (dd, *J* = 14.4, 9.7 Hz, 1H), 2.22 (s, 3H); ^13^C NMR (126 MHz, MeOD) δ 171.1, 156.4, 137.7, 130.5, 120.2, 78.4, 73.5, 64.9, 63.2, 56.7, 53.2, 52.6, 31.8, 20.9. HRMS (ESI^+^) calcd *m*/*z* for C_23_H_36_N_3_O_6_ [M + H]^+^ 450.2599, found 450.2602.

#### 2.5.10. (2*R*,3*S*,4*S*)-4-hydroxy-2-(4-iso-propoxybenzyl)-pyrrolidin-3-yl acetate (**22a**)

A mixture of hydroxybenzyl pyrrolidine **17** (93% pure, 11.7 mg, 0.046 mmol, 1 eq.) and saturated aq. NaHCO_3_ (0.3 mL) in THF (3 mL) at pH = 8–9 was treated with Boc_2_O (9.1 mg, 0.042 mmol, 1.1 eq.), stirred for 1 h, and concentrated in vacuo. The reduced volume was partitioned between EtOAc (5 mL) and water (2 mL). The organic phase was dried over MgSO_4_, filtered, and evaporated to a residue, which was purified by flash chromatography on silica gel eluting with a gradient of 60–90% EtOAc in hexane. Evaporation of the volatiles gave Boc-desmethylanisomycin **18** (9.7 mg, 0.028 mmol, 1 eq.), which was dissolved in DMF (1.5 mL) and treated with Cs_2_CO_3_ (9.1 mg, 0.028 mmol, 1 eq.) and 2-iodopropane (2.8 μL, 0.028 mmol, 1 eq.). The reaction mixture was heated to 65 °C, stirred for 2 h, and partitioned between EtOAc (5 mL) and water (2 mL). The aqueous phase was extracted with EtOAc (2 × 2 mL). The organic phases were combined, washed with brine (5 mL), dried over MgSO_4_, filtered, and concentrated to a residue that was purified by flash chromatography on silica gel eluting with a gradient of 40–90% EtOAc in hexane. Evaporation of the collected fractions gave the *N*-Boc-*O*-*iso*-propylanisomycin analog [7.7 mg, Rf = 0.34 (50% EtOAc in hexane)], which was dissolved in DCM (2 mL) and treated with HCl gas bubbles for 1 h. Evaporation of the volatiles under reduced pressure gave *iso*-propyl ether **22a** (6.3 mg, 69%), which was shown to be >99% pure by LC-MS analysis [20−80% MeOH (0.1% FA) in H_2_O (0.1% FA) over 14 min, RT 6.10 min]: [α]_D_^25^ 31.2 (*c* 0.17, MeOH); ^1^H NMR (500 MHz, MeOD) δ 8.53 (br s, 1H), 7.19 (d, *J* = 8.4 Hz, 2H), 6.88 (d, *J* = 8.5 Hz, 2H), 5.03 (br s, 1H), 4.57 (dt, *J* = 12.1, 6.1 Hz, 1H), 4.32 (d, *J* = 3.4 Hz, 1H), 4.11–4.08 (m, 1H), 3.59–3.55 (m, 1H), 3.15 (d, *J* = 12.5 Hz, 1H), 3.03 (dd, *J* = 14.0, 7.1 Hz, 1H), 2.98–2.91 (m, 1H), 2.17 (s, 3H), 1.29 (d, *J* = 6.0 Hz, 6H); ^13^C NMR (126 MHz, MeOD) δ 170.9, 158.6, 130.9, 129.2, 117.5, 78.6, 73.7, 71.0, 63.4, 52.6, 32.4, 22.3, 20.7. HRMS (ESI^+^) calcd *m*/*z* for C_16_H_24_NO_4_ [M + H]^+^ 294.1700, found 294.1692.

#### 2.5.11. (2*R*,3*S*,4*S*)-4-hydroxy-2-(4-octyloxybenzyl)-pyrrolidin-3-yl acetate (**22b**)

Employing the protocol for the synthesis of *iso*-propyl ether **22a**, residue **18** (8.3 mg, 0.024 mmol, 1 eq.) in DMF (1.5 mL) was treated with Cs_2_CO_3_ (7.8 mg, 0.024 mmol, 1 eq.) and 1-iodooctane (4.3 μL, 0.024 mmol, 1 eq.), worked up, and purified as described above to give octyl ether **22b** (6.2 mg, 65%), which was shown to be >95% pure by LC-MS analysis [30−95% MeOH (0.1% FA) in H_2_O (0.1% FA) over 14 min, RT 6.49 min]: [α]_D_^25^ 13.7 (*c* 0.16, MeOH); ^1^H NMR (500 MHz, MeOD) δ 7.22 (d, *J* = 8.7 Hz, 2H), 6.91 (d, *J* = 8.7 Hz, 2H), 5.07 (d, *J* = 3.3 Hz, 1H), 4.36 (d, *J* = 4.6 Hz, 1H), 4.17 (ddd, *J* = 9.8, 6.8, 3.3 Hz, 1H), 3.95 (t, *J* = 6.4 Hz, 2H), 3.60 (dd, *J* = 12.7, 4.4 Hz, 1H), 3.20 (d, *J* = 12.7 Hz, 1H), 3.08 (dd, *J* = 14.3, 6.7 Hz, 1H), 2.97 (dd, *J* = 14.2, 8.8 Hz, 1H), 2.19 (s, 3H), 1.79–1.72 (m, *J* = 14.4, 6.5 Hz, 2H), 1.50–1.43 (m, 2H), 1.37–1.29 (m, 8H), 0.91 (t, *J* = 7.0 Hz, 3H). NMR (126 MHz, MeOD) δ 170.8, 160.0, 130.9, 128.8, 116.1, 78.3, 73.4, 69.0, 63.7, 52.6, 33.0, 32.2, 30.5, 30.4, 30.3, 27.2, 23.7, 20.6, 14.4. HRMS (ESI^+^) calcd *m*/*z* for C_21_H_34_NO_4_ [M + H]^+^ 364.2482, found 364.2471.

#### 2.5.12. (2*R*,3*S*,4*S*)-4-hydroxy-2-(4-benzyloxybenzyl)-pyrrolidin-3-yl acetate (**22c**)

Employing the protocol for the synthesis of *iso*-propyl ether **22a**, residue **18** (9.5 mg, 0.027 mmol, 1 eq.) in DMF (1.5 mL) was treated with Cs_2_CO_3_ (8.8 mg, 0.027 mmol, 1 eq.) and benzyl bromide (3.2 μL, 0.027 mmol, 1 eq.), worked up, and purified as described above to give benzyl ether **22c** (7.4 mg, 73%), which was shown to be >99% pure by LC-MS analysis [20−80% MeOH (0.1% FA) in H_2_O (0.1% FA) over 14 min, RT 4.93 min]: [α]_D_^25^ 33.3 (*c* 0.20, MeOH); ^1^H NMR (500 MHz, MeOD) δ 8.54 (s, 1H), 7.44 (d, *J* = 7.4 Hz, 2H), 7.40–7.36 (m, *J* = 8.1, 6.7 Hz, 2H), 7.34–7.30 (m, 1H), 7.23 (d, *J* = 8.7 Hz, 2H), 7.01 (d, *J* = 8.7 Hz, 2H), 5.10 (s, 2H), 5.06 (d, *J* = 3.0 Hz, 1H), 4.35 (d, *J* = 4.6 Hz, 1H), 4.17–4.11 (m, 1H), 3.60 (dd, *J* = 12.7, 4.5 Hz, 1H), 3.18 (d, *J* = 12.7 Hz, 1H), 3.07 (dd, *J* = 14.2, 6.9 Hz, 1H), 3.00–2.95 (m, 1H), 2.19 (s, 3H); ^13^C NMR (126 MHz, MeOD) δ 170.9, 159.5, 138.7, 130.9, 129.6, 129.5, 128.9, 128.5, 116.5, 78.5, 73.6, 71.0, 63.4, 52.6, 32.4, 20.6. HRMS (ESI^+^) calcd *m*/*z* for C_20_H_23_NO_4_ [M + H]^+^ 342.1700, found 342.1696.

#### 2.5.13. (2*R*,3*S*,4*S*)-4-hydroxy-2-(4-carboxymethyloxybenzyl)-pyrrolidin-3-yl acetate (**22d**)

Employing the protocol used to prepare isopropyl ether **22a**, *N*-Boc-desmethylanisomycin **18** (8.6 mg, 0.025 mmol, 1 eq.) was dissolved in DMF (1.5 mL) and treated with Cs_2_CO_3_ (9.7 mg, 0.03 mmol, 1.2 eq.) and *tert*-butyl bromoacetate (4.4 μL, 0.03 mmol, 1 eq.). After stirring for 3 h, the volatiles were removed under vacuum. The residue (Rf = 0.48, 50% EtOAc in hexane) was dissolved in DCM (2 mL) and treated first with TFA (2 mL) for 30 min, followed by HCl gas bubbles for 1 h. Evaporation of the volatiles gave acid **22d** (6.1 mg, 62%), which was shown to be >99% pure by LC-MS analysis [10−90% MeOH (0.1% FA) in H_2_O (0.1% FA) over 14 min, RT 5.41 min]. ^1^H NMR (500 MHz, MeOD) δ 7.25 (d, *J* = 8.7 Hz, 2H), 6.95 (d, *J* = 8.7 Hz, 2H), 5.08 (d, *J* = 3.2 Hz, 1H), 4.66 (s, 2H), 4.36 (d, *J* = 4.6 Hz, 1H), 4.17 (dd, *J* = 5.9, 3.0 Hz, 1H), 3.60 (dd, *J* = 12.7, 4.4 Hz, 1H), 3.19 (d, *J* = 12.7 Hz, 1H), 3.10 (dd, *J* = 14.3, 6.5 Hz, 1H), 2.97 (dd, *J* = 14.3, 9.0 Hz, 1H), 2.18 (s, 3H). ^13^C NMR (126 MHz, CDCl_3_) δ 172.6, 170.8, 158.9, 131.0, 129.9, 116.3, 78.4, 73.4, 65.8, 63.6, 52.6, 32.2, 20.6. HRMS (ESI^+^) calcd *m*/*z* for C_15_H_20_NO_6_ [M + H]^+^ 310.1285, found 310.1280.

## 3. Results

### 3.1. Chemistry

**Scheme 1:**
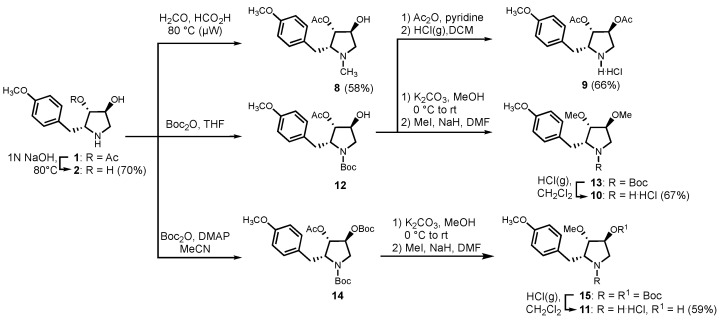
Synthesis of methylated anisomycin analogs.

Employing anisomycin (**1**) as a starting material, a set of amine and alcohol-modified analogs (e.g., **8**–**11**) were first synthesized to examine the importance of the polar substituents for anti-protozoan activity (Scheme 1). Desacetyl and diacetylanisomysin (**2** and **9**) were prepared to evaluate the relevance of the acyl functionality [38]. *N*-methyl anisomycin (**8**) has been previously synthesized by a fifteen-step synthesis from L-diethyl tartrate [39]. Employing anisomycin, *N*-methyl pyrrolidine **8** was prepared in 58% yield using Eschweiler–Clarke reaction conditions with formic acid and formaldehyde and microwave heating [40].

Modifications of the hydroxyl groups were accomplished by protocols commencing with *N*- and *N*,*O*-protection with the Boc group using di-*tert*-butyl dicarbonate in THF and in MeCN with 4-dimethylaminopyridine (DMAP) to give, respectively, *N*-(Boc)anisomycin (**12**) and *N*,*O*-bis-Boc-anisomycin (**14**) [41]. Subsequently, alcohol **12** was acylated using acetic anhydride in pyridine, and the Boc group was removed with HCl gas to furnish diacetylanisomycin (**9**) in 66% yield. *O*,*O*-bis-dimethyl ether **10** was also prepared from alcohol **12** in 67% yield by a route featuring saponification of the acetyl group, alkylation of the resulting diol with iodomethane and sodium hydride in DMF, followed by removal of the Boc group with HCl gas. Employing a similar protocol on *N*,*O*-bis-Boc-anisomycin (**14**) gave methyl ether **11** in 59% yield. 

**Scheme 2:**
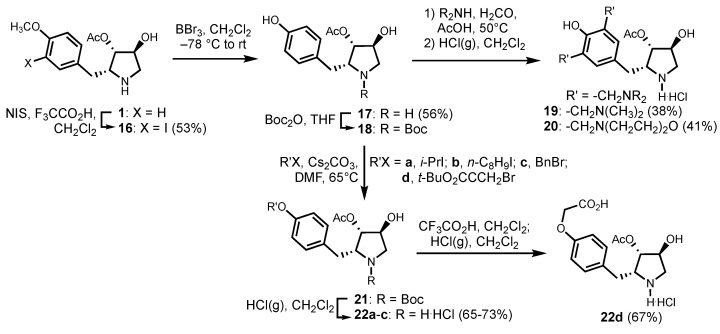
Synthesis of anisomycin analogs modified on the aromatic ring.

The aromatic ring of anisomycin (**1**) was next modified at the *meta*-position (e.g., **16**, **19**–**20**) by two different protocols (Scheme 2). Electrophilic iodination was accomplished with *N*-iodosuccinimide and trifluoroacetic acid in dichloromethane to provide iodide **16** in 53% yield. Substituents were also added to the aromatic ring of phenol **17**, which was obtained from anisomycin using boron tribromide in dichloromethane at –78 °C in 56% yield [42]. The amine of phenol **17** was protected using Boc_2_O in THF to provide *N*-Boc phenol **18**. Mannich reactions on phenol **18** using, respectively, dimethylamine and morpholine with formaldehyde provided bis-aminomethyl phenols **19** and **20** in 38% and 41% yields after Boc group removal with HCl gas. Moreover, phenol **18** was *O*-alkylated by alkoxide formation with cesium carbonate in DMF at 65 °C, followed by reaction with a set of alkyl halides: 2-iodopropane, 1-iodooctane, benzyl bromide, and *tert*-butyl bromoacetate. Ethers **22a**–**c** were prepared to respectively examine the effects of alkyl branching, long chain hydrophobicity, and aromaticity at the phenol position and obtained in 65–73% yields after Boc group removal with HCl gas. Carboxymethyl ether **22d** was isolated in 67% yield after Boc group and *tert*-butyl ester removal using trifluoroacetic acid in dichloromethane, followed by ion exchange with HCl gas to give the hydrochloride salt.

### 3.2. Anti-Leishmanial Activity of Anisomycin Derivatives 

Anisomycin and derivatives possessing modifications on the polar functional groups (e.g., **2** and **8**–**11**), aromatic *meta*-position (**16**, **19**, and **20**), and phenol (**17** and **22a**–**c**) were initially examined for inhibitory activity on protein synthesis in active lysates of *L. tarentolae*, *Escherichia coli*, and rabbit reticulocytes (Table 1). Anisomycin activity (IC_50_ = 0.55 μM) on *Leishmania* was typically eliminated by modification of the amine and alcohol functional groups (e.g., **2** and **8**–**10**). Notably, replacement of the 3-acetoxy group by a methyl ether gave a 44-fold drop in activity against *Leishmania* and a 700-fold loss in reticulocyte cytotoxicity, with a net improvement in the selectivity index. Furthermore, the introduction of substituents to the aromatic *meta*-position in analogs **16**, **19**, and **20** abolished anti-protozoan activity. Removal of the methyl ether at the aromatic *para*-position caused a nearly 100-fold loss of anti-protozoan activity, which was partially restored by replacing the methyl group with other ethers: *O-i*-Pr, *O*-*n*-octyl, and *O*-benzyl analogs **22a**–**c**. Ethers **22a**–**c** were, respectively, 2-, 67-, and 14-fold less active than anisomycin against *Leishmania*. Relative to the clinically used paromomycin, anisomycin (**1**) and *iso*-propyl ether **22a** exhibited better anti-protozoan activity, no effect on *E. coli*, but lower selectivity indices. Inhibitory activity on *L. donovani* promastigotes was subsequently measured according to the reported protocol [43] and reflected results from the inhibition of protein translation assay.

Considering the in vitro protein translation inhibition activities of the different ether analogs as well as activity on *L. donovani* prosmatigotes, the *O-i*-Pr, *O*-octyl, and *O*-carboxymethyl analogs (**22a**, **22c**, and **22d**) were further tested against promastigotes of various *Leishmania* strains, including *L. major* and *L. infantum*, as well as strains that developed resistance to current anti-leishmanial drugs such as pentavalent antimonials, miltefosine, and amphotericin B (Table 2). The most active *O*-*i*-Pr analog, **22a**, in the translation assay exhibited similar inhibitory activity against the different *Leishmania* promastigotes and slightly improved activity against the resistant strains as that displayed by anisomycin (**1**).

**Figure 2:**
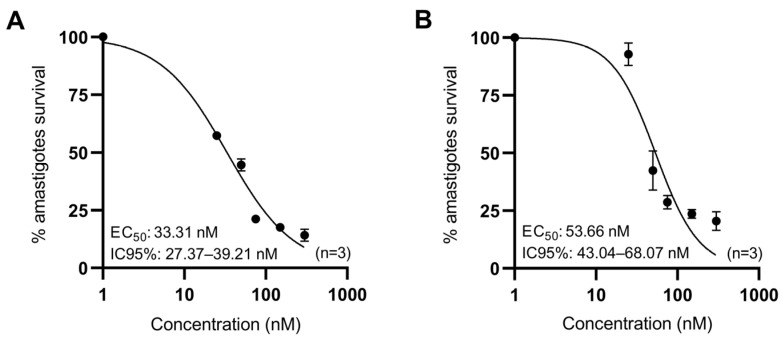
Effect of *O-iso*-propyl ether **22a** on BMDM macrophages infected with (**A**) *L. infantum* WT and (**B**) *L. major* WT for 48 h.

The most active analog in the promastigote assays, *O-iso*-propyl ether **22a**, was tested on intracellular forms of *Leishmania* in macrophages. Against the amastigotes of *L. infantum* and *L. major*, ether **22a** exhibited, respectively, 3.3 and 3.6-fold higher activity (EC_50_ 33.31 nM and 53.66 nM) than that observed against the promastigote (Figure 2). In addition, ether **22a** exhibited 5–10-fold greater selectivity against the different amastigotes relative to host macrophage toxicity, which was examined using a 3-(4,5-dimethylthiazol-2-yl)-2,5-diphenyl-tetrazolium bromide (MTT) survival assay. 

## 4. Discussion

Focused on a better understanding of the structure–activity relationships of anisomycin against *Leishmania*, a systematic study was performed to validate the importance of the pyrrolidine and aromatic ring substituents for anti-parasitic activity and reticulocyte toxicity.

**Figure 3:**
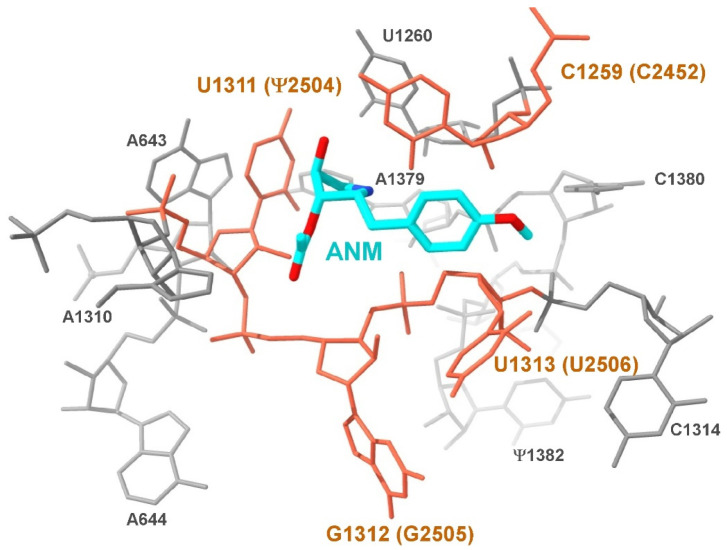
Graphical depiction of the conserved binding site of anisomycin in *L. major* ribosome. The 80S ribosome (8OVJ) of *L. major* was superimposed on the 50S ribosome of *H. marismortui* (PDB ID: 3CC4), and nucleotides proposed to contact anisomycin (colored in cyan) are highlighted in orange; the corresponding homologs in *E. coli* rRNA are presented in parentheses; pseudouridine nucleotide is indicated with Ψ symbol.

Previously, crystal structures of anisomycin bound to eukaryotic ribosomes (*Saccharomyces cerevisiae*) and archaeal ribosomes (*Haloarcula marismortui*) demonstrated high conservation in the binding orientation [4]. Anisomycin is positioned in the peptidyl transferase center of the ribosome through the hydrogen bonds formed by the hydroxy group (with nucleotides of LSU rRNA Y2504, G2505 (*E. coli* numbering)) and the nitrogen (C2452) [44]. Considering that the binding site of anisomycin is highly conserved between bacteria, yeast, humans, and *Leishmania*, a graphic model was constructed to rationalize the observed results (Figure 3). 

Decreases in anti-leishmanial activity upon modification of the pyrrolidine ring substituents may likely be due to losses of key hydrogen-bond interactions. For example, deacetyl, 3-methoxy, and 3,4-dimethoxy analogs **2**, **10**, and **11** were inactive against *L. tarentolae* translation and confirmed the importance of the acetyl group as previously reported using an anti-yeast assay [45]. The affinity and specificity of anisomycin for the ribosome A-site are also determined by the interactions of the aromatic ring with the nucleotide bases of C2452 and U2506 [43]. The loss of activity of the mono- and di-*meta*-substituted analogs (e.g., **20**) suggests that steric hindrance may prevent the entrance of aromatic rings in the A-site. 

Upon binding to the ribosome, the *p*-methoxyphenyl group inserts completely into the hydrophobic crevice of the A-site and blocks the access of the incoming aminoacyl-tRNAs, causing the disruption of peptide bond formation [46]. The inactivity of the demethylated analog on the inhibition of translation may be explained by a loss of hydrophobicity. On the other hand, alternative phenolic ethers were tolerated. Notably, *O*-*i*-Pr, *O*-Octyl, *O*-benzyl, and *O*-CH_2_CO_2_H phenolic ethers **22a**–**22d** all retained varying degrees of anti-parasitic activity in the *Leishmania* translation assay and against live promastigotes. Moreover, certain phenolic ethers (e.g., *O*-*i*-Pr **22a**) exhibited subtle but significantly lower inhibition of reticulocyte translation, indicating the potential to differentiate toxicity from anti-leishmanial activity. Against the intracellular parasite stage (amastigotes), the anti-leishmanial activity of *O*-*i*-Pr **22a** was higher than against the extracellular stage (promastigotes) [47]. This phenomenon demonstrated that *O*-*i*-Pr **22a** was able to enter the host BMDM cells to act on the intracellular amastigote infection without host cell toxicity [48]. 

## 5. Conclusions

The anti-protozoal activity of anisomycin has been known since the 1950s. In spite of intriguing mechanisms of action involving inhibitory activity on protein synthesis and modulatory activity on various kinases, the therapeutic utility of anisomycin has been compromised due to its limited selectivity. A systematic study of the importance of the pyrrolidine and aromatic ring functional groups for the anti-protozoal activity of anisomycin has revealed a high sensitivity to chemical modification. Earlier substitutions of the *para*-methoxy substituent by methyl and protons gave analogs with reduced anti-protozoal activity [16]. Conversion of the methoxyphenyl group to alternative ethers has, however, illuminated an entry point for designing anisomycin analogs that differentiate anti-leishmanial activity from host cytotoxicity. Future efforts to improve anti-parasitic potency and selectivity are under investigation and will be reported in due time.

## Figures and Tables

**Table 1:** Inhibition of translation in active cell lysates derived from *L. tarentolae*, bacteria (*E. coli*), and rabbit reticulocytes (RR) and inhibition of *L. donovani* promastigote growth by anisomycin and derivatives.

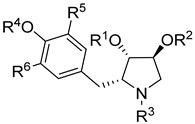											
Analog	R^1^	R^2^	R^3^	R^4^	R^5^	R^6^	IC_50_ (μM)			LC_50_ (μM)	SI
							*L. tarentolae*	*E. coli*	RR		
Paromomycin							3.62 ± 0.09	0.051 ± 0.005	21 ± 1.1	35.2	0.2
**1**	Ac	H	H	Me	H	H	0.55 ± 0.03	>500	0.1 ± 0.02	0.22 ± 0.015	2.5
**2**	H	H	H	Me	H	H	>100	>1000	>1000	>>600	-
**8**	Ac	H	Me	Me	H	H	>100	>1000	25.5 ±3.6	>>600	-
**9**	Ac	Ac	H	Me	H	H	>100	>1000	25.7 ± 8.5	>>600	-
**10**	Me	Me	H	Me	H	H	>1000	>1000	>1000	>>600	-
**11**	Me	H	H	Me	H	H	24 ± 2.5	>1000	70.9 ± 8.3	>>600	0.3
**16**	Ac	H	H	Me	I	H	>1000	>1000	73.7 ± 1.7	>>600	-
**17**	Ac	H	H	H	H	H	53.7 ± 6.1	>200	6.2 ± 0.45	35±7.2	8.6
**19**	Ac	H	H	H	-CH_2_N(CH_3_)_2_		>1000	>200	70.7 ± 7.3	>>600	-
**20**	Ac	H	H	H	-CH_2_N(CH_2_CH_2_)_2_O		>1000	>1000	>1000	>>600	-
**22a**	Ac	H	H	*i*-Pr	H	H	1.13 ± 0.12	>200	0.9 ± 0.3	0.3 ± 0.12	1.2
**22b**	Ac	H	H	*n*-octyl	H	H	33.5 ± 8.2	>200	9.5 ± 3.5	5.2 ± 1.5	3.5
**22c**	Ac	H	H	Bn	H	H	7.96 ± 1.02	>200	0.9 ± 0.2	1.9 ± 0.3	8.8

IC_50_: inhibition of translation. LC_50_: inhibition of *Leishmania donovani* (MHOM/SD/1962/1S-Cl2d) promastigotes growth. SI (selectivity index) = IC_50_ *L. tarentolae*/IC_50_ RR.

**Table 2:** Anti-leishmanial activity of anisomycin and derivatives.

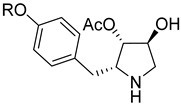						
#	R	IC_50_ (μM)				
		Ldi WT ^1^	LmF WT ^2^	Ldi Sb-Res ^3^	Ldi MF-Res ^4^	Ldi AmB-Res ^5^
**Miltefosine**		7.16 ± 0.21	8.47 ± 1.18	8.21 ± 0.53	>200	30.34 ± 4.28
**Antimony**		75.80 ± 6.13	25.02 ± 1.13	>2000	85.33 ± 7.10	90.25 ± 8.30
**Anisomycin** (**1**, nM)		123.9 ± 5.34	109.56 ± 9.16	294.9 ± 12.45	467.5 ± 30.11	168.6 ± 10.90
**22a** (nM)	*i*-Pr	121.80 ± 5.62	180.17 ± 4.69	128.80 ± 3.16	132.70 ± 7.80	118.90 ± 14.07
**22b**	*n*-Octyl	1.11 ± 0.03	1.75 ± 0.12	1.75 ± 0.08	1.83 ± 0.06	1.78 ± 0.58
**22d**	-CH_2_CO_2_H	14.9 ± 2.18	16.55 ± 2.23	15.98 ± 1.03	18.17 ± 2.33	19.23 ± 1.24

^1^ *Leishmania infantum* (MHOM/MA/67/ITMAP-263) wild-type strain; ^2^ Leishmania major Friedlin; ^3^ *Leishmania infantum* (MHOM/MA/67/ITMAP-263) resistant to 2 mM trivalent (and pentavalent) antimonials; ^4^ *Leishmania infantum* (MHOM/MA/67/ITMAP-263) resistant to 200 µM miltefosine; ^5^ *Leishmania infantum* (MHOM/MA/67/ITMAP-263) resistant to 1 µM amphotericin B.

## Data Availability

Not applicanle.
